# Subregional structural and connectivity damage in the visual cortex in neuromyelitis optica

**DOI:** 10.1038/srep41914

**Published:** 2017-02-03

**Authors:** Huanhuan Cai, Jiajia Zhu, Ningnannan Zhang, Qiuhui Wang, Chao Zhang, Chunsheng Yang, Jie Sun, Xianting Sun, Li Yang, Chunshui Yu

**Affiliations:** 1 Department of Radiology and Tianjin Key Laboratory of Functional Imaging, Tianjin Medical University General Hospital, Tianjin 300052, China; 2 Department of Neurology, Tianjin Medical University General Hospital, Tianjin 300052, China

## Abstract

Patients with neuromyelitis optica (NMO) have shown structural and functional impairments in the visual cortex. We aimed to characterize subregional grey matter volume (GMV) and resting-state functional connectivity (rsFC) changes in the visual cortex in NMO. Thirty-seven NMO patients and forty-two controls underwent structural and functional MRI scans. The GMV and rsFC of each visual subregion were compared between the groups. Compared with controls, NMO patients had GMV reductions in the bilateral V1, V2, V3d, VP, and LO and in the left V3A. In canonical visual pathways, the relatively low-level subregions showed more significant GMV reductions than did the high-level ones. Regardless of GMV correction, NMO patients showed reduced rsFC in the bilateral LO and V4v and in the left V2. The GMVs of the bilateral V1 and LO and of the left V2 and V3d were negatively correlated with clinical disability in NMO patients; these correlation coefficients were associated with hierarchical positions in the visual pathways. These findings suggest that in NMO, the low-level visual subregions have more severe structural damage; structural damage is not the only factor affecting rsFC alterations of visual subregions; GMV reduction in the low-level visual subregions has the highest predictive value for clinical disability.

Neuromyelitis optica (NMO) is characterized by optic nerve damage that may result in structural and functional alterations of the posterior visual pathways via Wallerian degeneration^1^,^2^,^3^,^4^. For example, NMO patients have shown reduced grey matter volume (GMV)^2^,^5^ and altered spontaneous brain activity^6^,^7^ in the visual cortex and impaired integrity in the optic radiation^3^,^4^,^5^,^8^,^9^,^10^,^11^,^12^,^13^,^14^.

The visual cortex is a heterogeneous region and has been subdivided into distinct areas; a visual area is defined as a swath of cortex that has similar function, architectonics, connections and topography^15^. However, the structural and functional damage of the visual cortex in NMO at the subregional level remains largely unknown. Investigating the structural and functional damage of the visual subregions in NMO is clinically important because doing so can improve our understanding of the neural mechanisms underlying the visual cortex impairment in NMO by answering the following questions: (1) whether the structural damage in the visual subregions is associated with the hierarchical positions in the visual pathways in NMO; (2) whether the structurally impaired visual subregions are also functionally impaired in NMO; and (3) which measure and which visual subregion can better predict clinical disability in NMO patients.

To answer these questions, we performed an exploratory study by collecting structural MRI and resting-state functional MRI data from 37 NMO patients and 42 healthy controls. Using the GMV as a measure of structural damage and the resting-state functional connectivity (rsFC) as a measure of functional damage, we compared the GMV and rsFC differences in each visual subregion between the groups.

## Methods

### Study participants

This study was approved by the Medical Research Ethics Committee at Tianjin Medical University General Hospital, and after complete description of the study to the participants, written informed consent was obtained. The methods were carried out in accordance with the approved guidelines. We recruited a total of 37 NMO patients and 42 healthy controls. These two groups did not show any significant differences in sex, age, or education years (Table 1). All patients satisfied the revised Wingerchuk diagnostic criteria for NMO^16^, including the two absolute criteria of optic neuritis and myelitis and at least two of the following three additional criteria: brain MRI negative or nondiagnostic for multiple sclerosis at onset, MRI evidence of a spinal cord lesion of three or more vertebral segments, and a serological test positive for NMO antibodies. The exclusion criteria were MRI contraindications, history of head trauma or other neuropsychiatric diseases, other autoimmune diseases, and poor image quality. The disease severity of patients was assessed by the Expanded Disability Status Scale (EDSS) scores. The EDSS is a widely used scale for quantifying the level of overall disability in patients with multiple sclerosis and NMO. The EDSS scale ranges from 0 to 10 in 0.5 unit increments. A higher score indicates a more severe overall disability.

### MRI data acquisition

MRI data were acquired using a 3.0-Tesla MR scanner (Discovery MR750, General Electric, Milwaukee, WI, USA). Tight but comfortable foam padding was used to minimize head motion, and earplugs were used to reduce scanner noise. Sagittal 3D T1-weighted images were acquired by a brain volume sequence with the following parameters: repetition time (TR) = 8.2 ms; echo time (TE) = 3.2 ms; inversion time (TI) = 450 ms; flip angle (FA) = 12°; field of view (FOV) = 256 mm × 256 mm; matrix = 256 × 256; slice thickness = 1 mm, no gap; and 188 sagittal slices. Resting-state fMRI data were acquired using a gradient-echo single-short echo planar imaging sequence: TR/TE = 2000/45 ms; FOV = 220 mm × 220 mm; matrix = 64 × 64; FA = 90°; slice thickness = 4 mm; gap = 0.5 mm; 32 interleaved transverse slices; and 180 volumes. All subjects were instructed to keep their eyes closed, relax, move as little as possible, think of nothing in particular, and not fall asleep during the fMRI scans. All images were visually inspected to ensure that only images without visible artifacts were included in subsequent analyses. Conventional brain and spinal cord MRIs were also performed to detect visible lesions.

### GMV calculation

The GMV of each voxel was calculated using the VBM8 toolbox (http://dbm.neuro.uni-jena.de/vbm.html). Structural MRI images were segmented into grey matter (GM), white matter and cerebrospinal fluid using the standard segmentation model. After an initial affine registration of the GM concentration map into Montreal Neurological Institute (MNI) space, GM concentration images were nonlinearly warped using diffeomorphic anatomical registration through the exponentiated Lie algebra (DARTEL) technique, and the results were resampled to a voxel size of 3 × 3 × 3 mm^3^. The relative GMV of each voxel was obtained by multiplying the GM concentration map by the non-linear determinants derived from the spatial normalization. Finally, the GMV images were smoothed using a Gaussian kernel of 6 mm × 6 mm × 6 mm full-width at half-maximum (FWHM). We used Gaussian kernel to smooth the data for two reasons: (1) to improve the signal to noise ratio and thereby make the structural MRI data satisfy a Gaussian distribution, which is the prerequisite for parametric statistics, and (2) to further overcome the alignment problem in the spatial normalization step. After spatial preprocessing, the smoothed GMV maps were used for statistical analyses.

### Preprocessing for fMRI data

The fMRI data were preprocessed using the Data Processing Assistant for Resting-State fMRI Advanced Edition (DPARSFA)^17^ and Statistical Parametric Mapping (SPM8, http://wwwfil.ion.ucl.ac.uk/spm). The first 10 volumes of each subject were discarded to allow the signal to reach equilibrium and the participant to adapt to the scanning noise. The remaining volumes were corrected for the acquisition time delay between slices. Then, realignment was performed to correct for head motion between time points. All subjects’ fMRI data were within the defined motion thresholds (i.e., translational or rotational motion parameters less than 2 mm or 2°). We also calculated the frame-wise displacement (FD), which indexes the volume-to-volume changes in head position. Several nuisance covariates (six motion parameters, their first time derivations, and average blood-oxygenation-level-dependent (BOLD) signals of the ventricular, white matter and whole brain) were regressed out from the data. Recent studies have reported that the signal spike caused by head motion significantly contaminated the final resting-state fMRI results even after regressing out the linear motion parameters^18^. Therefore, we further regressed out spike volumes when the FD of the specific volume exceeded 0.5. In the normalization step, individual structural images were linearly co-registered with the mean functional image; the structural images were then nonlinearly transformed to MNI space. Then, the transformation parameters were applied to the functional images. The functional images were resampled into a 3 × 3 × 3 mm^3^ voxel and smoothed with a Gaussian kernel of 6 mm FWHM. After linear detrending, the functional images were band-pass filtered with a frequency range of 0.01 to 0.08 Hz^19^.

### Definition of the visual subregions

**Figure 1:**
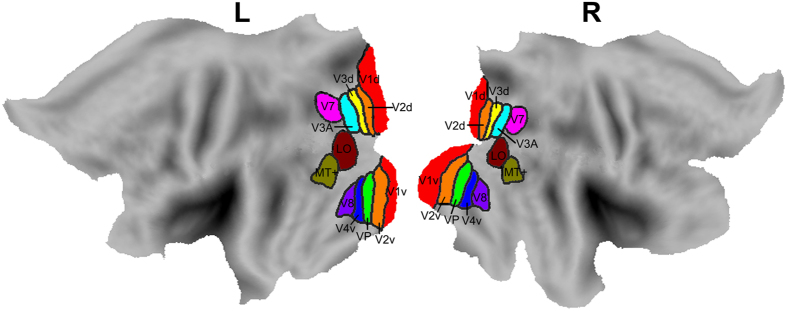
The locations of the visual subregions. L: left; R: right.

The visual subregions were defined according to a previous study that has subdivided the visual cortex in each hemisphere into 10 areas, i.e., the LO, MT, V1, V2, V3A, V3d, V4v, V7, V8 and VP^15^,^20^. These visual subregions are shown in Fig. 1.

### GMV differences of the visual subregions

After these visual subregions were normalized into the MNI space, we calculated the GMV of each visual subregion for each group and compared the values obtained for the two groups. Multiple comparisons were corrected using the Bonferroni method (*p* < 0.05/20 = 0.0025).

To quantitatively compare GMV reduction among visual subregions in NMO, we calculated the ratio of GMV reduction in each visual subregion for each NMO patient using the following equation: GMV reduction ratio = (mean GMV of controls – GMV of each patient)/mean GMV of controls. Using this equation, we can obtain the GMV reduction of each visual subregion in each patient relative to the mean GMV of that subregion in healthy controls. Then, we used repeated measures analysis of variance (ANOVA) to test whether there was a significant difference in GMV reduction among the visual subregions in each hemisphere in NMO patients (*p* < 0.05). If the differences were significant, we used a pair-wise *t*-test to compare GMV reduction between every two visual subregions (*p* < 0.05). To explore the association between GMV reduction and hierarchical positions in the visual pathways, we investigated differences in GMV reduction in NMO in the canonical visual pathways, including the dorsal (V1, V2, V3d, V3A, and V7) and ventral (V1, V2, VP, V4v, and V8) visual pathways in each hemisphere (*p* < 0.05). The hierarchical position of a visual subregion was ordered by its location in the dorsal or ventral visual pathway. The hierarchical positions of visual subregions in the dorsal visual pathway from low to high are: 1 = V1, 2 = V2, 3 = V3d, 4 = V3A and 5 = V7; and the hierarchical positions of visual subregions in the ventral visual pathway from low to high are: 1 = V1, 2 = V2, 3 = VP, 4 = V4v and 5 = V8. We used a linear mixed effect model to investigate the association between the hierarchical position in the canonical visual pathways and the GMV reduction in NMO patients. The random intercept term accounts for the correlation due to repeated measurements within a single subject. The model considered hierarchical position as a fixed effect and subject as a random effect. The model was expressed as the following equation:


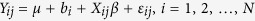


where *Y*_*ij*_ is the GMV reduction of the *j*th visual subregion of the *i*th patient (*i* ≦ 37, *j* ≦ 16); *μ* is the intercept term that is common to all visual subregions; *b*_*i*_ accounts for average GMV reduction across subregions of the *i*th patient; *X*_*ij*_ is the hierarchical position (ranged from 1 to 5) of the *j*th visual subregion of the *i*th patient; *β* is the estimated parameter of the fixed effect (i.e., the hierarchical position); and *ε*_*ij*_ is the residual error of the model. The model parameters were estimated by the restricted maximum likelihood method and considered significant if the *p* value was less than 0.05. Futhermore, we made a slope between GMV reduction and hierarchical positions of visual subregions in each NMO patient, and then tested whether the slope was different from 0. The slope of each patient is the Spearman correlation coefficient between GMV reduction and hierarchical positions of visual subregions in the visual pathways in this NMO patient.

### Voxel-wise rsFC analysis

For each participant, Pearson’s correlation coefficients between the mean time series of each visual subregion and the time series of each voxel in other parts of the brain were computed and converted to z-values using Fisher’s r-to-z transformation to improve the normality^21^. Then, individuals’ z-values were entered into a random-effect one-sample *t*-test in a voxel-wise manner using the SPM8. A family-wise error (FWE) correction with *p* < 0.05 was used to identify brain regions that showed significant positive correlations with each subregion. Then, a two-sample *t*-test was performed within the positive connectivity mask to quantitatively compare the differences in rsFC in each subregion between the two groups, controlling for age, sex and years of education. A voxel-wise false discovery rate (FDR) correction with *p* < 0.05 and a minimum cluster size of >30 voxels was used to identify the statistical significance. To investigate the effect of GMV atrophy of each visual subregion on its corresponding rsFC changes, we extracted the average rsFC of the voxels within each cluster which showed significant rsFC difference with this subregion between the groups. Then, we used a general linear model to compare the intergroup differences in these rsFCs with and without GMV correction.

### Statistical analysis

The demographic and clinical data were analyzed using the Statistical Package for the Social Sciences version 19.0 (SPSS, Chicago, IL, USA). To test whether the GMV or the rsFC of the visual subregions with significant group differences were correlated with clinical variables, we extracted these measures and calculated partial correlation coefficients controlling for age, sex and years of education between these measures and EDSS scores. Multiple comparisons were corrected using the Bonferroni method (*p* < 0.05/27 = 0.0019).

## Results

### Demographic and clinical characteristics of participants

The demographic and clinical data of NMO patients and healthy controls are listed in Table 1. Compared with healthy controls, patients with NMO did not show any significant differences in age (*t* = 0.831, *p* = 0.409), sex (χ^2^ = 0.046, *p* = 0.830) or years of education (*t* = 1.049, *p* = 0.297). The mean EDSS score in the patient group was 3.9 ± 2.5.

### The GMV differences in the visual subregions

**Figure 2:**
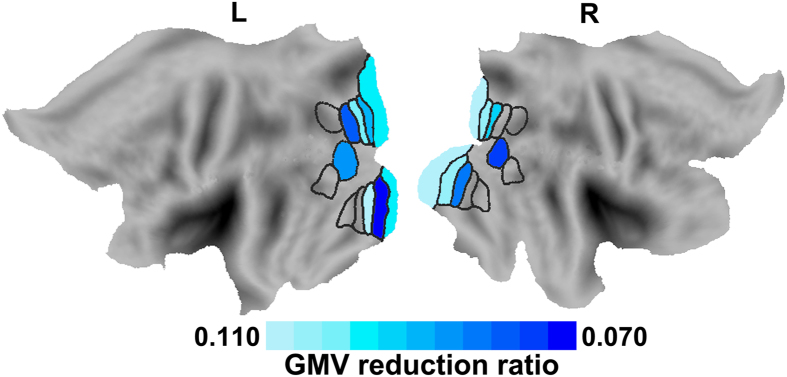
Visual subregions with significant GMV differences between NMO patients and healthy controls (*p* < 0.05, Bonferroni corrected). Color represents the ratio of GMV reduction in each visual subregion in NMO patients. The ratio of GMV reduction in each visual subregion in NMO was calculated by the following equation: GMV reduction ratio = (mean GMV of controls − mean GMV of patients)/mean GMV of controls. Only visual regions with significant GMV reduction were marked with colors. GMV: grey matter volume; L: left; NMO: neuromyelitis optica; R: right.

Compared with healthy controls, NMO patients had significantly reduced GMV in the bilateral LO, V1, V2, V3d and VP and in the left V3A (*p* < 0.05/20 = 0.0025, Bonferroni corrected) (Fig. 2, Supplementary Fig. S1 and Table S1). The repeated measures ANOVA showed significant differences in GMV reduction among visual subregions in both hemispheres (left: *F* = 6.549, *df* = 44, *p* < 0.001; right: *F* = 5.948, *df* = 44, *p* < 0.001) in NMO patients. The GMV reduction across visual subregions in NMO is shown in Supplementary Fig. S2. In the canonical visual pathways, we found that the relatively low-level subregions showed a greater GMV reduction in NMO than did the relatively high-level ones. Specifically, the V1 and V3d showed a greater GMV reduction than did the V7 in the left dorsal visual pathway (Supplementary Fig. S2B); the V1, V2 and V3d showed a greater GMV reduction than did the V3A, and the V1 had a greater GMV reduction than did the V7 in the right dorsal pathway (Supplementary Fig. S2E). The V1, V2 and VP had a greater GMV reduction than did the V4v and V8 in the bilateral ventral pathways (Supplementary Fig. S2C and F). The linear mixed effect model analysis revealed a significant negative association (*β* = −0.203, *t* = −7.477, *p* < 0.001) between GMV reduction and hierarchical positions of the visual subregions. The mean slope between GMV reduction and hierarchical positions in NMO patients were −0.321 ± 0.405, which was significantly lower than 0 (*one-sample t-test, t* = −4.823, *p* < 0.001) (Supplementary Fig. S3).

### The GMV differences in other cortical regions

We also compared GMV differences in the other cortical areas (e.g., the primary motor, somatosensory and auditory areas) and found that these regions also showed significant GMV reduction in NMO patients compared to healthy controls (Supplementary Table S2).

### The rsFC patterns in the visual subregions

The rsFC maps of the left and right visual subregions are shown in Supplementary Figs S4 and S5, respectively. Both healthy controls and NMO patients exhibited similar rsFC patterns for each visual subregion, which was mainly correlated with brain regions in the occipital cortex; however, the patient group showed a smaller spatial extent than the control group.

### The rsFC differences in the visual subregions

**Figure 3:**
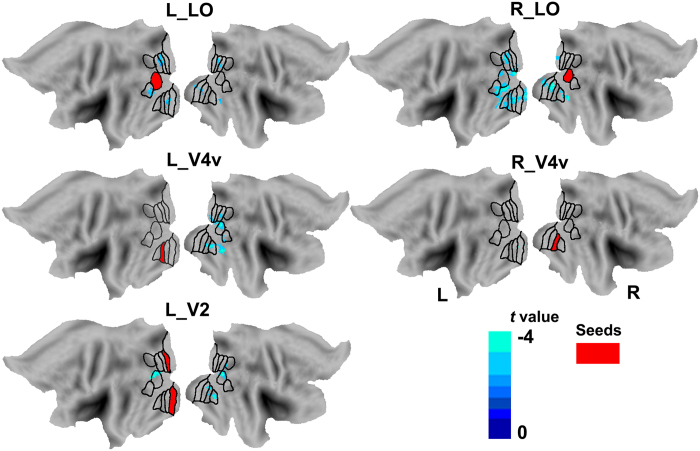
Brain regions with significant rsFC differences with the visual subregions between NMO patients and healthy controls (*p* < 0.05, FDR corrected). Red color represents the seed region. Blue color indicates reduced rsFC in NMO. FDR: false discovery rate; L: left; NMO: neuromyelitis optica; R: right; rsFC: resting-state functional connectivity.

Compared with healthy controls, NMO patients showed significantly reduced rsFC in the bilateral LO and V4v and in the left V2 (*p* < 0.05, FDR corrected) (Fig. 3). In NMO patients, the left LO had decreased rsFC with the bilateral V2, V3d, V3A and VP, with the left MT, and with the right V1 and temporal cortex. The left V2 had decreased rsFC with the bilateral LO and with the right V4v, V7, V8 and VP. The left V4v had decreased rsFC with the right LO, MT, V1, V2, V3d, V3A, V4v, V8, VP and temporal cortex. The right LO had decreased rsFC with the bilateral V1, V2, V4v, V8, VP and temporal cortex and with the left LO, MT, V3d and V3A. The right V4v had decreased rsFC with the left V1, V2 and VP. Neither reduced rsFC in other visual subregions nor increased rsFC were observed in any subregion in NMO patients (*p* < 0.05, FDR corrected).

### The rsFC differences in the visual subregions after GMV correction

To test whether the rsFC changes in a visual subregion are secondary to GMV reduction in this subregion, we compared intergroup rsFC differences of this subregion with and without GMV correction. All of the rsFCs with significant intergroup differences were still significant after GMV correction (Supplementary Table S3).

### Clinical correlations

**Figure 4:**
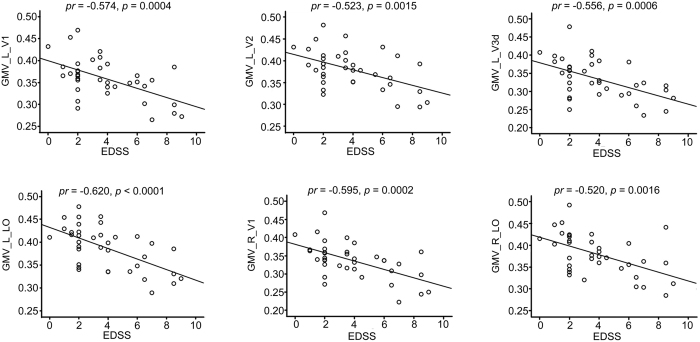
Significant correlations between the GMV of visual subregions and the EDSS scores in NMO patients (*p* < 0.05, Bonferroni corrected). EDSS: Expanded Disability Status Scale; GMV: grey matter volume; L: left; NMO: neuromyelitis optica; R: right.

**Figure 5:**
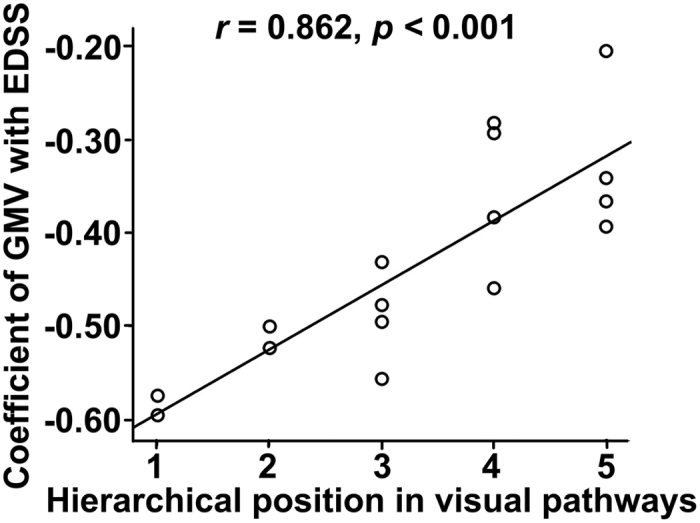
Spearman correlations between the hierarchical position in the visual pathways and the correlation coefficient of the GMV of each visual subregion with EDSS scores in NMO patients. The hierarchical position of a visual subregion was ordered by its location in the dorsal or ventral visual pathway. The hierarchical positions of visual subregions in the dorsal visual pathway from low to high are: 1 = V1, 2 = V2, 3 = V3d, 4 = V3A and 5 = V7; and the hierarchical positions of visual subregions in the ventral visual pathway from low to high are: 1 = V1, 2 = V2, 3 = VP, 4 = V4v and 5 = V8. In the x axis, 1 represents the bilateral V1; 2 represents the bilateral V2; 3 represents the bilateral V3d and VP, 4 represents the bilateral V3A and V4v; and 5 represents the bilateral V7 and V8. EDSS: Expanded Disability Status Scale; GMV: grey matter volume; NMO: neuromyelitis optica.

In NMO patients, partial correlations (controlling for age, sex and years of education) were performed between the EDSS scores and the GMVs of the 11 significant subregions and the rsFCs of 16 clusters showing significant intergroup differences (*p* < 0.05/27 = 0.0019, Bonferroni corrected). The EDSS scores were negatively correlated with the GMVs of the left V1 (*pr* = −0.574, *p = *0.0004), V2 (*pr* = −0.523, *p* = 0.0015) and V3d (*pr* = −0.556, *p = *0.0006), LO (*pr* = −0.620, *p* < 0.0001) and with the GMV of the right V1 (*pr* = −0.595, *p* = 0.0002) and LO (*pr* = −0.520, *p* = 0.0016) (Fig. 4, Supplementary Table S4). No significant correlations were found between the EDSS scores and the GMV or rsFC of any other visual subregions (Supplementary Table S4). Moreover, we found a significant positive correlation between the correlation coefficients of GMV with EDSS and hierarchical positions of the visual subregions (Fig. 5).

## Discussion

In accordance with previous studies reporting GMV reduction^2^,^5^ and cortical thinning^22^,^23^, we found a GMV reduction in the visual cortex in NMO, which suggested that structural damage in the visual cortex is a stable feature of NMO. Beyond prior studies, we found that GMV reduction in visual subregions was associated with their hierarchical positions in the canonical visual pathways. The relatively low-level visual subregions (e.g., V1 and V2) have more severe structural damage than the relatively high-level ones (e.g., V7 and V8). The structural damage in the visual cortex in NMO has been explained by the following mechanisms: Wallerian degeneration secondary to the damage of the optic nerves^2^,^3^,^4^,^5^,^8^,^14^; axonal degeneration secondary to the white matter damage of the brain^2^,^8^,^9^,^11^,^12^,^13^; and occult damage in the visual cortex^24^,^25^. Only the first mechanism can predict more severe structural damage in the relatively low-level visual areas than in the high-level ones because the relatively low-level visual areas are more densely connected with the optic nerves and are expected to be more severely damaged by the Wallerian degeneration process. Thus, our findings suggest that Wallerian degeneration secondary to the optic nerve damage is an important neural mechanism for the structural damage in the visual cortex in NMO. However, these findings cannot exclude the existence of other neural mechanisms that may contribute to the structural damage in the visual cortex because some putative high-level visual subregions (such as the LO, V3d and VP) also showed GMV reduction in NMO. Moreover, the GMV reduction in the auditory cortex further supports the idea that other neural mechanisms may be involved in cortical atrophy in NMO because neither the optic nerve nor the spinal cord directly connects to the auditory cortex.

Resting-state fMRI has been used to explore the functional alterations in the brain in NMO and revealed reduced ALFF and increased synchronization in the visual cortex^6^,^7^. The rsFC measures the temporal coherence of BOLD signals between every two spatially remote regions or voxels and has been thought to reflect functional integration of the brain^26^,^27^,^28^. The rsFC reduction is mainly restricted to the visual cortex and the visual pathways, which suggests that there is functional integration impairment in the visual network in NMO. Moreover, both the inter- and intra-hemispheric rsFCs were impaired, indicating that both the inter- and intra-hemispheric functional integration of the visual network is impaired in NMO. We found that the rsFC alterations remained significant, even with GMV correction, suggesting that GMV reduction in a visual subregion is not the only factor that leads to rsFC alterations of this visual subregion. In addition to factors affecting the GMV of the visual cortex, the rsFC of the visual cortex in NMO may be affected by several other factors, including the structural and functional changes in the connected regions, the dependence of subregions on the visual input, and the functional reorganization. Because multiple factors might be associated with rsFC reduction in the visual cortex in NMO, the specific contribution of these factors should be clarified in future studies. For the voxel-wise rsFC analysis, 20 visual subregions were used as seeds in identical analyses, which may increase the probability of false positives. One possibility would be to take alpha = 0.05/20 = 0.0025 to correct for extra comparisons. However, no cluster can pass this strict correction in our analysis. Thus, we cannot rule out the possibility that the resulting effects are due to false positives. In future studies, a larger sample is needed to validate our findings.

We found that the GMVs of several visual subregions were negatively correlated with EDSS scores in NMO patients, thus suggesting a potential role of GMV reduction of these visual subregions in predicting the clinical disability in NMO. Moreover, we found significant correlations between these correlation coefficients and hierarchical positions in the visual pathways. These findings suggest that GMV reductions in the low-level visual subregions have higher predictive value for clinical disability in NMO patients than do GMV reductions in the high-level subregions. However, no rsFCs of any visual subregions were correlated with EDSS scores, reducing the possibility of using the rsFCs of visual subregions to predict the clinical severity in NMO. The discrepancy in the clinical predictive values of the GMV and rsFC of visual subregions may be associated with the differences in neural mechanisms that result in the GMV and rsFC impairments. The GMV reductions in these low-level visual subregions in NMO are mainly caused by Wallerian degeneration secondary to the damage of the optic nerves. However, the rsFC reduction in the visual cortex results from the combined effects of multiple mechanisms.

In summary, we found that the relatively low-level visual subregions had greater GMV reduction than the relatively high-level ones in NMO, suggesting that Wallerian degeneration secondary to optic nerve damage is an important mechanism for structural damage in the visual cortex. We also found spatial inconsistency in the visual subregions with weaker rsFCs and smaller GMVs, and there was no significant effect of GMV reduction on the rsFC changes in any visual subregions in NMO. These findings indicate that structural damage is not the only factor accounting for rsFC alterations in visual subregions. The GMVs of several visual subregions were correlated with clinical disability in NMO patients, suggesting that they are potential biomarkers for predicting clinical disability in NMO.

## Additional Information

**How to cite this article**: Cai, H. *et al*. Subregional structural and connectivity damage in the visual cortex in neuromyelitis optica. *Sci. Rep.* **7**, 41914; doi: 10.1038/srep41914 (2017).

**Publisher's note:** Springer Nature remains neutral with regard to jurisdictional claims in published maps and institutional affiliations.

## Supplementary Material

Supplementary Materials

## Figures and Tables

**Table 1:** Demographic and clinical characteristics of the participants.

	NMO patients (n = 37)	Healthy controls (n = 42)
Sex, F/M	32/5	37/5
Age, y, Mean ± SD	46.97 ± 14.28	49.19 ± 9.17
Years of education, Mean ± SD	10.73 ± 3.95	11.55 ± 2.96
EDSS score, Mean ± SD	3.9 ± 2.5	NA

EDSS: Expanded Disability Status Scale; NMO: neuromyelitis optica; NA: not applicable.
