# Supplementary material for: Subregional structural and connectivity damage in the visual cortex in neuromyelitis optica
Source: Sci Rep. 2017 Feb 3;7:41914. doi: 10.1038/srep41914 (PMC5291226; doi:10.1038/srep41914)
Supplement: Supplementary Materials [file srep41914-s1.pdf]

Title: Subregional structural and connectivity damage in the visual cortex in neuromyelitis optica

Huanhuan Cai, MS[1†]; Jiajia Zhu, PhD[1†]; Ningnannan Zhang, PhD[1]; Qiuhui Wang, MD[1]; Chao Zhang, MD[2]; Chunsheng Yang, MD[2]; Jie Sun, MS[1]; Xianting Sun, MS[1]; Li Yang, MD[2]; Chunshui Yu, MD[1*]

[1]Department of Radiology and Tianjin Key Laboratory of Functional Imaging, and [2]Department of Neurology, Tianjin Medical University General Hospital, Tianjin 300052, China.

[†]These authors contributed equally to the manuscript.

* Correspondence and requests for materials should be addressed to Chunshui Yu, Department of Radiology, Tianjin Medical University General Hospital, No. 154, Anshan Road, Heping District, Tianjin 300052, China

E-mail: chunshuiyu@tijmu.edu.cn

Phone: +86-22-63062026

Fax: +86-22-63062290

Methods

GMV differences of other cortical regions. We extracted the bilateral precentral gyrus, postcentral gyrus, Heschl's gyrus and superior temporal gyrus from automated anatomical labeling (AAL) template, and calculated the GMV of each cortical region and compared it between the two groups.

Results

The GMV differences in other cortical regions. Compared with healthy controls, NMO patients showed significantly reduced GMV in the bilateral precentral gyrus, postcentral gyrus, Heschl's gyrus and superior temporal gyrus ($p < 0.05$) (Supplementary Table S2).

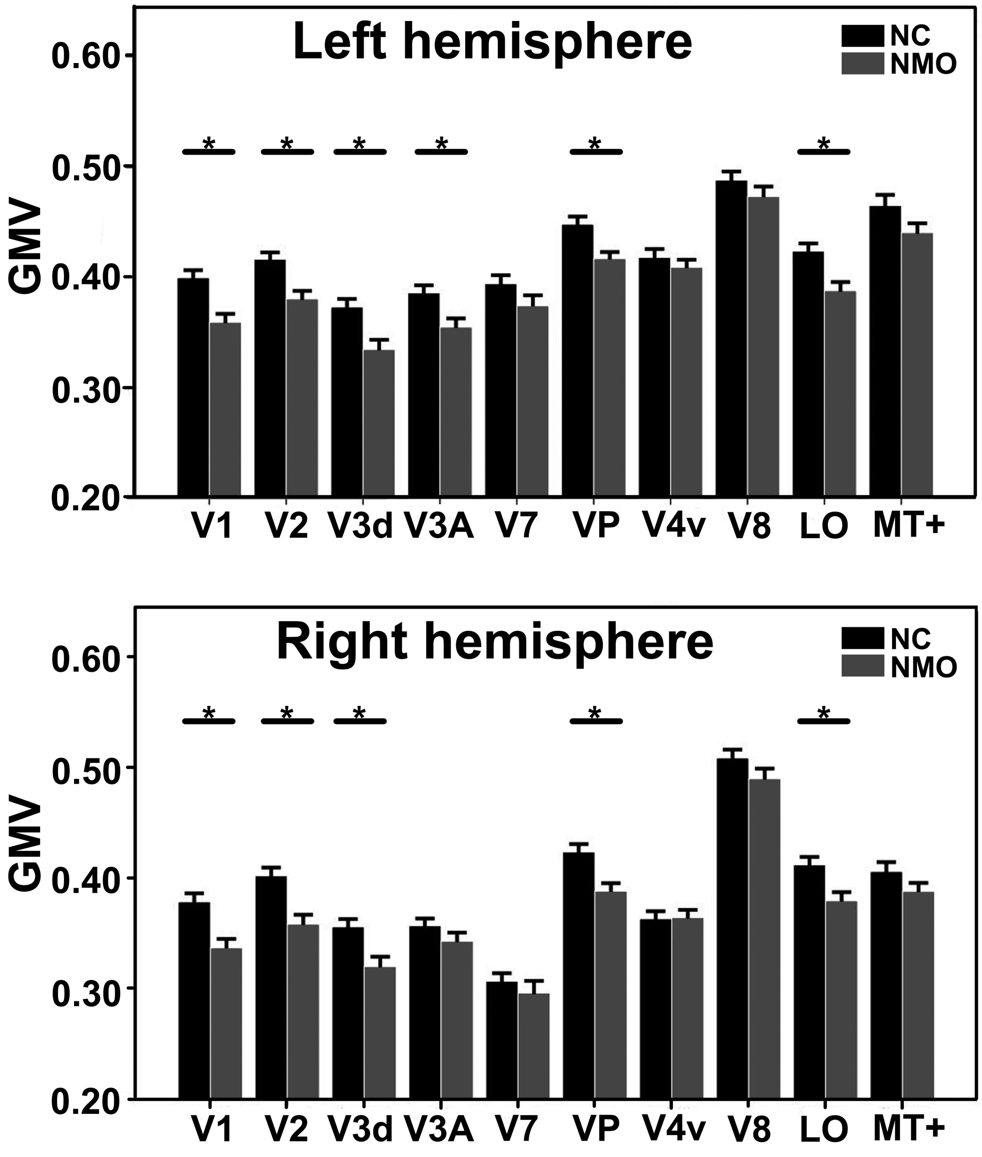

Supplementary Figure S1. Bar graph shows GMV difference in each visual subregion between NMO patients and healthy controls. The error bar is the standard error. GMV: grey matter volume; NC: normal control; NMO: neuromyelitis optica; *p < 0.05, Bonferroni corrected.

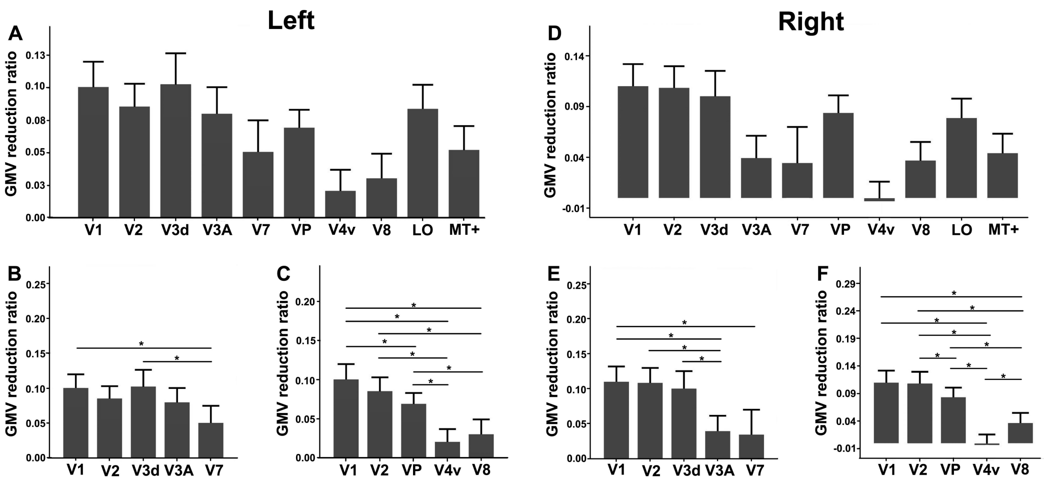

Supplementary Figure S2. GMV reduction across visual subregions in NMO. (A) GMV reduction in visual subregions of the left hemisphere in NMO; (B) GMV reduction across visual subregions in the left dorsal visual pathway; and (C) GMV reduction across visual subregions in the left ventral visual pathway; (D) GMV reduction in visual subregions of the right hemisphere in NMO; (E) GMV reduction across visual subregions in the right dorsal visual pathway; and (F) GMV reduction across visual subregions in the right ventral visual pathway. Error bars are standard errors. GMV: grey matter volume; NMO, neuromyelitis optica; *$p < 0.05$.

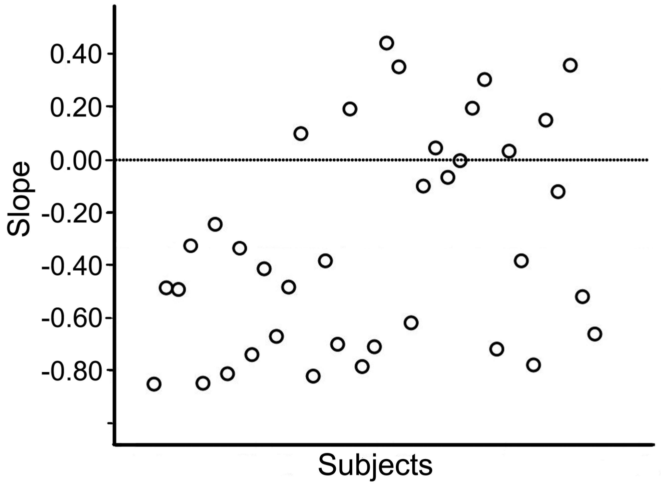

Supplementary Figure S3. Scatter plot of slopes between GMV reduction and hierarchical positions of visual subregions in NMO patients. The slope of each patient is the Spearman correlation coefficient between GMV reduction and hierarchical positions of visual subregions in the visual pathways in this NMO patient. The horizontal line denotes zero.

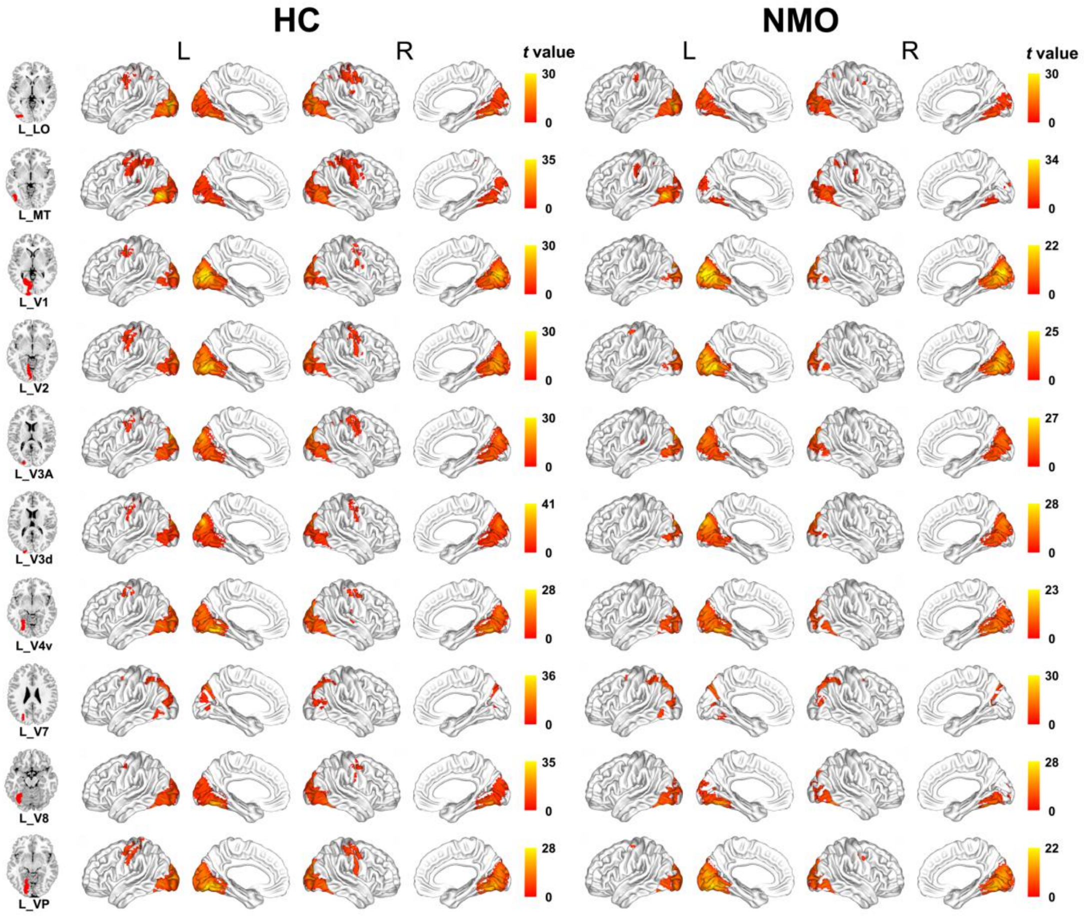

Supplementary Figure S4. The rsFC maps of the left visual subregions in NMO patients and healthy controls ($p < 0.05$, FWE corrected). Only positive rsFCs of each visual subregion are depicted. FWE: family-wise error; HC: healthy controls; L: left; NMO: neuromyelitis optica; R: right; rsFC: resting-state functional connectivity.

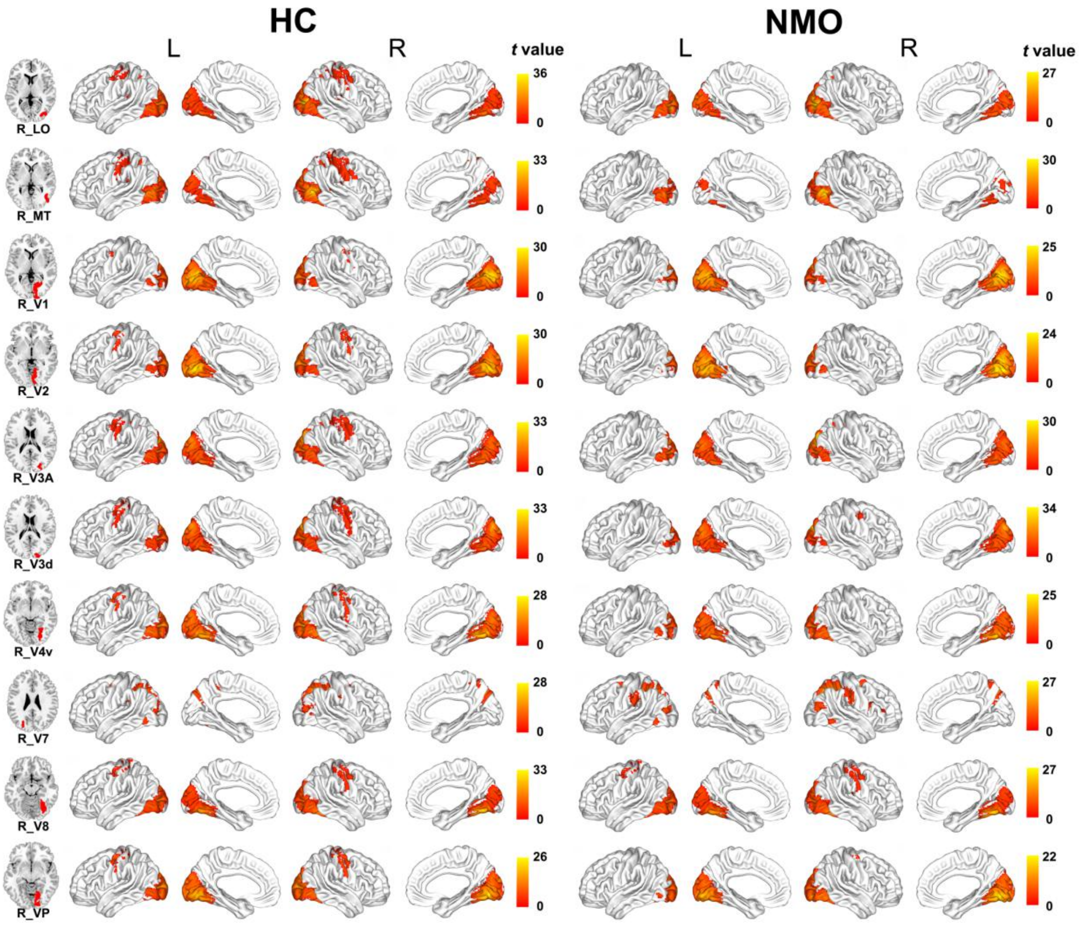

Supplementary Figure S5. The rsFC maps of the right visual subregions in NMO patients and healthy controls ($p < 0.05$, FWE corrected). Only positive rsFCs of each visual subregion are depicted. FWE: family-wise error; HC: healthy controls; L: left; NMO: neuromyelitis optica; R: right; rsFC: resting-state functional connectivity.

Supplementary Table S1. GMV differences in the visual subregions between NMO patients and healthy controls (p < 0.05/20 = 0.0025, Bonferroni corrected)

Visual subregions	NMO patients (n=37)		Healthy controls (n=42)		Statistics	
	Mean	SD	Mean	SD	*F*	*p* values
L_LO	0.388	0.047	0.423	0.045	16.033	<0.001*
L_MT	0.440	0.051	0.465	0.063	5.675	0.020
L_V1	0.359	0.047	0.400	0.045	19.701	<0.001*
L_V2	0.380	0.044	0.416	0.041	21.030	<0.001*
L_V3A	0.355	0.048	0.386	0.044	13.217	0.001*
L_V3d	0.335	0.054	0.373	0.047	16.924	<0.001*
L_V4v	0.409	0.041	0.418	0.049	1.861	0.177
L_V7	0.374	0.058	0.394	0.049	3.972	0.050
L_V8	0.473	0.056	0.488	0.050	4.163	0.045
L_VP	0.417	0.037	0.448	0.045	19.387	<0.001*
R_LO	0.380	0.048	0.412	0.046	13.071	0.001*
R_MT	0.388	0.047	0.406	0.055	3.822	0.054
R_V1	0.337	0.050	0.379	0.050	18.784	<0.001*
R_V2	0.358	0.052	0.402	0.049	21.381	<0.001*
R_V3A	0.343	0.047	0.357	0.041	3.306	0.073
R_V3d	0.320	0.054	0.356	0.459	14.872	<0.001*
R_V4v	0.364	0.043	0.363	0.045	0.004	0.951
R_V7	0.296	0.066	0.307	0.047	1.098	0.298
R_V8	0.490	0.056	0.509	0.492	5.153	0.026
R_VP	0.388	0.044	0.424	0.456	19.980	<0.001*

* Significant intergroup differences. Abbreviations: GMV: gray matter volume; NMO: neuromyelitis optica.

Supplementary Table S2. GMV differences in other cortical regions between NMO patients and healthy controls.

Cortical areas*	NMO patients (n=37)		Healthy controls (n=42)		Statistics	
	Mean	SD	Mean	SD	*F*	*p* values
L_Pre_CG	0.288	0.044	0.306	0.035	10.078	0.002*
R_Pre_CG	0.291	0.044	0.307	0.035	7.668	0.007*
L_Post_CG	0.287	0.048	0.300	0.036	5.227	0.025*
R_Post_CG	0.276	0.045	0.295	0.038	8.458	0.005*
L_HG	0.429	0.072	0.461	0.059	10.879	<0.001*
R_HG	0.408	0.063	0.442	0.062	11.167	<0.001*
L_ STG	0.394	0.055	0.417	0.048	10.278	0.002*
R_ STG	0.371	0.045	0.391	0.041	9.479	0.003*

*These cortical areas are extracted from AAL template.

AAL, automated anatomical labeling; GMV, grey matter volume; HG, Heschl gyrus; L, left; NMO, neuromyelitis optica; Post_CG, postcentral gyrus; Pre_CG, precentral gyrus; R, right; SD, standard deviation; STG, superior temporal gyrus.

Supplementary Table S3. The rsFC differences of the visual subregions between NMO patients and healthy controls before and after GMV correction

Connected regions	Before GMV correction	After GMV correction
L_LO-B_Cun	14.607(<0.001)*	10.285(0.002)*
L_LO-L_IOG	19.446(<0.001)*	18.380(<0.001)*
L_LO-L_LG	16.037(<0.001)*	13.467(<0.001)*
L_LO-R_FG/ITG	18.933(<0.001)*	17.719(<0.001)*
L_LO-R_LG	14.319(<0.001)*	13.142(0.001)*
L_V2-L_MOG	16.171(<0.001)*	11.835(0.001)*
L_V2-R_LG/FG	22.195(<0.001)*	19.068(<0.001)*
L_V2-R_MOG	23.979(<0.001)*	15.983(<0.001)*
L_V4v-R_FG/ITG	25.748(<0.001)*	24.077(<0.001)*
L_V4v-R_MOG/SOG	18.098(<0.001)*	17.338(<0.001)*
L_V4v-R_IOG/ITG	11.483(0.001)*	11.462(0.001)*
R_LO-L_LG/FG	25.080(<0.001)*	22.051(<0.001)*
R_LO-L_MOG	12.922(0.001)*	12.489(0.001)*
R_LO-L_SOG/Cun	11.682(0.001)*	11.144(0.001)*
R_LO-R_LG/FG	24.653(<0.001)*	19.578(<0.001)*
R_V4v-L_LG	19.402(<0.001)*	19.593(<0.001)*

Note. The data are shown as the F value (p value). * $p < 0.05$. Abbreviation: B: bilateral; Cun: cuneus; FG: fusiform gyrus; GMV: grey matter volume; IOG: inferior occipital gyrus; ITG: inferior temporal gyrus; L: left; LG: lingual gyrus; MOG: middle occipital gyrus; NMO: neuromyelitis optica; R: right; rsFC: resting-state functional connectivity; SOG: superior occipital gyrus.

Supplementary Table S4. Correlations between imaging and clinical parameters

Imaging measures	*pr*	*p*
GMV		
L_LO	-0.620	<0.0001*
L_V1	-0.574	0.0004*
L_V2	-0.523	0.0015*
L_V3A	-0.459	0.0063
L_V3d	-0.556	0.0006*
L_VP	-0.477	0.0043
R_LO	-0.520	0.0016*
R_V1	-0.595	0.0002*
R_V2	-0.500	0.0027
R_V3d	-0.495	0.0029
R_VP	-0.431	0.0109
rsFC		
L_LO-B_Cun	-0.221	0.2098
L_LO-L_IOG	-0.064	0.7190
L_LO-L_LG	0.044	0.8057
L_LO-R_FG/ITG	-0.311	0.0733
L_LO-R_LG	0.151	0.3952
L_V2-L_MOG	-0.202	0.2528
L_V2-R_LG/FG	-0.191	0.2792
L_V2-R_MOG	-0.185	0.2937
L_V4v-R_FG/ITG	-0.263	0.1335
L_V4v-R_MOG/SOG	-0.297	0.0885
L_V4v-R_IOG/ITG	-0.099	0.5771
R_LO-L_LG/FG	-0.308	0.0763
R_LO-L_MOG	-0.220	0.2105

R_LO-L_SOG/Cun	-0.236	0.1786
R_LO-R_LG/FG	-0.235	0.1818
R_V4v-L_LG	-0.351	0.0417

Note. The data are shown as the partial correlation coefficient and *p* value.

* Multiple comparisons were corrected using the Bonferroni method ($p < 0.05/27 = 0.0019$).

Abbreviation: B: bilateral; Cun: cuneus; EDSS: Expanded Disability Status Scale; FG: fusiform gyrus; GMV: grey matter volume; IOG: inferior occipital gyrus; ITG: inferior temporal gyrus; L: left; LG: lingual gyrus; MOG: middle occipital gyrus; NMO: neuromyelitis optica; R: right; rsFC: resting-state functional connectivity; SOG: superior occipital gyrus.
